# Catheter-related blood stream infection caused by *Raoultella ornithinolytica*

**DOI:** 10.1007/s12223-015-0390-2

**Published:** 2015-04-19

**Authors:** Alicja Sękowska, Katarzyna Dylewska, Eugenia Gospodarek, Tomasz Bogiel

**Affiliations:** Department of Microbiology, Nicolaus Copernicus University in Toruń, Ludwik Rydygier Collegium Medicum in Bydgoszcz, 9 M. Skłodowskiej-Curie Street, 85-094 Bydgoszcz, Poland; Department of Paediatric, Hematology and Oncology, Nicolaus Copernicus University in Toruń, Ludwik Rydygier Collegium Medicum in Bydgoszcz, 9 M. Skłodowskiej-Curie Street, 85-094 Bydgoszcz, Poland

## Abstract

*Raoultella* spp. representatives are Gram-negative capsulated, nonmotile rods. These bacteria are found in the natural environment: plants, water, soil and insects. *R. ornithinolytica* is one of the three species of *Raoultella. R. ornithinolytica* is the only species within the genus which has the ability to produce ornithine decarboxylase. Human infections related to *R. ornithinolytica* are exceedingly rare. The present case report describes catheter-related blood stream infection caused by *R. ornithinolytica* and successfully treated with antibiotic therapy.

## Introduction

*Raoultella* species are Gram-negative nonmotile rods belonging to *Enterobacteriaceae* family. Initially, *Raoultella* spp. were classified as *Klebsiella* spp., but on the basis of 16S ribosomal RNA and *rpoB* gene sequences, these two genera were discriminated (Drancourt et al. 2001). These bacteria are found in the natural environment and in animals. Human infections related to *Raoultella* spp. are exceedingly rare. *Raoultella ornithinolytica* was initially described as *Klebsiella ornithinolytica* by Sakazaki et al. in 1989 (Sakazaki et al. 1989). The first human infection caused by *R. ornithinolytica* was reported in 2009 (Morais et al. 2009; Vos and Laureys 2009). However, the correct identification of *Raoultella* species is very difficult. By the conventional methods used routinely in the microbiological laboratory, *Raoultella* species are mostly indistinguishable from *Klebsiella pneumoniae* and *Klebsiella oxytoca*.

## Case report

An 8-year-old child attended the Dr Antoni Jurasz University Hospital in Bydgoszcz with a fever. She had a history of brain tumour (*retinoblastoma*), a neurogenic bladder and frequent urinary tract infections. On admission to the paediatric clinic, the following laboratory tests were done: the peripheral blood counts: WBC—12.63 × 10^3^/μL, RBC—3.82 × 10^6^/μL, HGB—11.0 g/dL, MCV—83.5 fL and HCT—31.9 %. The procalcitonin level was 12 mg/L, and C-reactive protein (CRP) level was 159 mg/L. In view of the fever (38.9 °C), blood was taken routinely, as well as from a Broviac catheter, the latter both for microbiology tests and for blood culture. The tunnelled catheter was inserted 69 days before. Because of a neurogenic bladder, a urine sample was also collected and cultured.

After 8 h and 49 min, a positive culture from the catheter-derived blood was obtained; while after 11 h and 19 min, a positive culture was also obtained. Both blood cultures yielded *R. ornithinolytica*. The strains demonstrated a mucoid colony on blood agar and mucoid pale pink colony on MacConkey agar. The strains were identified with an automated system: VITEK2 Compact (bioMérieux, biotype 4437711753066431, at a probability level of 87.0 %). The identification of the isolates was further confirmed using the technique called Matrix-Assisted Laser Desorption Ionization-Time of Flight Mass Spectrometry (MALDI-TOF MS) (Bruker, Germany). The assay was done twice to yield four scores: three for *R. ornithinolytica* scores—2.14-2.46-2.48 (medium 2.36) and one for *R. planticola*—2.02. Thus, the obtained score (≥2.300) indicated the correct identification at the species level.

*R. ornithinolytica* strains were sensitive to piperacillin, ticarcillin with clavulanic acid, piperacillin with tazobactam, cefuroxime, cefotaxime, ceftazidime, cefepime, imipenem, meropenem, amikacin, tobramycin, netilmicin, ciprofloxacin and cotrimoxazole. The strains were resistant to ampicillin and amoxicillin with clavulanic acid. The urine culture grew *Proteus mirabilis* sensitive to the following antibiotics: piperacillin, ticarcillin with clavulanic acid, piperacillin with tazobactam, cefuroxime, cefotaxime, ceftazidime, cefepime, imipenem, meropenem, amikacin, tobramycin, netilmicin, ciprofloxacin and cotrimoxazole. None of the cultured isolates produced extended-spectrum beta-lactamases. The child does not have any therapeutic agents at the time of acquiring the infection, neither has a rash.

The patient received courses of piperacillin with tazobactam (4 × 3.5 g, i.v.) and amikacin (1 × 500 mg, i.v.) for 6 days. The procalcitonin level on the next day was 43 mg/L with CRP 261 mg/L and 192 mg/L on the third day. On the fourth day, both the blood culture and urine culture were negative; on the fifth day, CRP was 36 mg/L. On the tenth day, both, the blood and urine samples, were taken for the follow-up control. The cultures were negative; the catheter was sterile, and the patient recovered. It suggests usefulness of the treatment applied.

## Discussion

Rarely are Gram-negative rods isolated in catheter-related blood stream infections (CRBSI) (Nielsen et al. 2012). Catheter-related blood stream infections are usually caused by coagulase-positive staphylococci, especially *Staphylococcus aureus*, and coagulase-negative staphylococci. *Raoultella* spp. rods are primarily found in water, soil and plants. *R. ornithinolytica* was isolated from fish, ticks and termites.

Our case illustrates a CRBSI with a Gram-negative rod. Since *R. ornithinolytica* was first described in 2009 (Morais et al. 2009; Vos and Laureys 2009), only several cases of clinical infections in humans have been reported and none of catheter-related infection. Morais et al. identified the case of an enteric fever-like syndrome caused by *R. ornithinolytica* in an 82-year-old patient with a history of arterial hypertension and degenerative arthropathy. The patient was treated with amoxicillin with clavulanic acid. At the same time, Vos and Laureys reported colic distension associated with an inflammatory syndrome in a 97-year-old woman. The patient had a giant renal cyst. The woman was not treated with antibiotics, but the inflammatory syndrome resolved on its own. The third patient was an infant with visceral heterotaxy (Mau and Ross 2010). The child showed skin flushing, and the authors identified *R. ornithinolytica*. In 2011, Solak et al. (2011) identified *R. ornithinolytica* in a 44-year-old woman with diabetic foot lesion and a history of diabetes mellitus, hypertension, hypothyroidism and chronic kidney disease. The patient also had a rash on her legs. The patient was treated initially with piperacillin and tazobactam, and then with tigecycline, because of multidrug resistance. Some authors reported bacteraemia (Hadano et al. 2012) and urinary tract infections (García-Lozano et al. 2013) in cancer patients.

*R. ornithinolytica* are Gram-negative rods rarely found in human infections. Correct identification of these bacteria is still a challenge as they are possibly still being misidentified as *Klebsiella* on the basis of histamine production; many of the histamine-producing strains were identified as *K. pneumoniae* or *K. oxytoca* at first, but additional tests or methods later enabled their correct identification as *Raoultella* rods. Thus, *R. planticola* and *R. ornithinolytica* are histamine-producing bacteria as opposed to members of genus *Klebsiella*. The use of additional tests allows for the differentiation of these two bacterial genera. Rods of *Raoultella* genus can use histamine while *Klebsiella* ethanolamine as the only source of carbon in the medium. Moreover, *Raoultella* genus use 3-dl-beta-hydroxybutyric acid as the only source of carbon in the medium, but this property is also characteristic to selected *Klebsiella* strains. Species of *R. planticola* and *R. ornithinolytica* can grow at 4 °C. *R. ornithinolytica* representatives produce ornithine decarboxylase and *R. terrigena* decompose of d-melezitose.

The MALDI-TOF MS technique is a rapid, simple and high-throughput proteomic system which appears to have been useful in bacteria identification (Van Veen et al. 2010). This is a soft ionization technique used in mass spectrometry, allowing the analysis of biomolecules (such as DNA, proteins and sugars) and large organic molecules (such as polymers and dendrimers), which tend to be fragile and fragmented when ionized by more conventional ionization methods. The MALDI-TOF MS technique demonstrated high sensitivity and specificity for *R. ornithinolytica*. The obtained score (≥2.300) indicated the correct identification at the species level; while 2.000–2.299 at the genus level and probable at the species level, 1.700–1.999 indicated the probable identification at the genus level. This technique can be used for rapid identification of various microorganisms: aerobic and anaerobic bacteria, Gram-positive and Gram-negative bacteria as well as mycobacteria and yeasts.

*Raoultella* strains are usually sensitive to antibiotics (amoxicillin with clavulanic acid, piperacillin, piperacillin with tazobactam, cefuroxime, cefotaxime, ceftazidime, cefepime, imipenem, meropenem, ertapenem, gentamicin, amikacin, tobramycin, netilmicin, ciprofloxacin, levofloxacin, tigecycline and cotrimoxazole). However, Castanheira et al. (2009) described three strains of *Raoultella* spp. (two *R. planticola* and one *R. ornithinolytica*) isolated from patients; resistant to carbapenems, the drugs of choice when infections of multidrug resistant Gram-negative strains appear.

Our case report describes catheter-related bacteraemia caused by *R. ornithinolytica*, which was successfully treated with the applied antibiotic therapy (piperacillin with tazobactam and amikacin). As *Raoultella* spp. infections in humans are rare, the full implications of *Raoultella* as a human pathogen remain unknown.
